# Selective logging impacts on soil microbial communities and functioning in Bornean tropical forest

**DOI:** 10.3389/fmicb.2024.1447999

**Published:** 2024-09-26

**Authors:** Samuel J. B. Robinson, Dafydd M. O. Elias, Tim Goodall, Andrew T. Nottingham, Niall P. McNamara, Robert Griffiths, Noreen Majalap, Nicholas J. Ostle

**Affiliations:** ^1^UK Centre for Ecology & Hydrology, Lancaster, United Kingdom; ^2^Lancaster Environment Centre, Lancaster University, Lancaster, United Kingdom; ^3^UK Centre for Ecology & Hydrology, Wallingford, United Kingdom; ^4^School of Geography, University of Leeds, Leeds, United Kingdom; ^5^Smithsonian Tropical Research Institute, Ancón, Panama; ^6^School of Natural Sciences, Bangor University, Bangor, United Kingdom; ^7^Forest Research Centre, Sabah Forestry Department, Sandakan, Malaysia

**Keywords:** soil bacteria, soil fungi, soil biogeochemical cycling, soil heterotrophic respiration, soil enzymes, canopy gap, dipterocarp

## Abstract

Rainforests provide vital ecosystem services that are underpinned by plant–soil interactions. The forests of Borneo are globally important reservoirs of biodiversity and carbon, but a significant proportion of the forest that remains after large-scale agricultural conversion has been extensively modified due to timber harvest. We have limited understanding of how selective logging affects ecosystem functions including biogeochemical cycles driven by soil microbes. In this study, we sampled soil from logging gaps and co-located intact lowland dipterocarp rainforest in Borneo. We characterised soil bacterial and fungal communities and physicochemical properties and determined soil functioning in terms of enzyme activity, nutrient supply rates, and microbial heterotrophic respiration. Soil microbial biomass, alpha diversity, and most soil properties and functions were resistant to logging. However, we found logging significantly shifted soil bacterial and fungal community composition, reduced the abundance of ectomycorrhizal fungi, increased the abundance of arbuscular mycorrhizal fungi, and reduced soil inorganic phosphorous concentration and nitrate supply rate, suggesting some downregulation of nutrient cycling. Within gaps, canopy openness was negatively related to ectomycorrhizal abundance and phosphomonoesterase activity and positively related to ammonium supply rate, suggesting control on soil phosphorus and nitrogen cycles via functional shifts in fungal communities. We found some evidence for reduced soil heterotrophic respiration with greater logging disturbance. Overall, our results demonstrate that while many soil microbial community attributes, soil properties, and functions may be resistant to selective logging, logging can significantly impact the composition and abundance of key soil microbial groups linked to the regulation of vital nutrient and carbon cycles in tropical forests.

## 1 Introduction

Tropical forests are the most biologically rich and ecologically complex ecosystems on the planet (Laurance, 2007), playing a central role in global biogeochemical cycling and carbon (C) storage (DeFries et al., 2002; Zarin, 2012). These essential ecosystem functions are driven by complex plant–soil interactions and reciprocal feedbacks between aboveground vegetation and belowground soil microbial communities (Bever et al., 2010; Cortois et al., 2016; van der Heijden et al., 2008; van der Putten et al., 2013; Wardle et al., 2004). The rainforests of Borneo are globally significant reservoirs of biodiversity and C (Myers et al., 2000; Qie et al., 2017) but are under increasing pressure from rapid degradation and loss due to commercial timber extraction and widespread conversion to plantation agriculture (Asner et al., 2018; Bryan et al., 2013; Ferraz et al., 2018; Gaveau et al., 2016; Gaveau et al., 2014; Qie et al., 2017). Over 30% of the original forest cover on Borneo has been lost since the early 1970s, with approximately 45% (16.8 Mha) of the remaining forest in 2015 modified by selective logging (Gaveau et al., 2016), having major downstream consequences for forest C cycling (Riutta et al., 2018).

The lowland rainforests of Borneo are characterised by canopy dominance of dipterocarp trees (Whitmore, 1984) which, due to their high commercial value as timber, are primarily targeted during selective logging operations (Appanah and Turnbull, 1998). The removal of individual dipterocarp trees and the creation of landings and skid trails opens gaps in the forest canopy, leaving a significant proportion of the remaining forest a highly heterogeneous mosaic of closed canopy (hereafter “intact”) forest and selective logging gaps (hereafter “logging gaps”) at various stages of regeneration (Bossel and Krieger, 1991; Ellis et al., 2016). LiDAR survey in Malaysian Borneo has shown logging can increase the prevalence of gaps 15-fold compared to old-growth forest, raising the canopy gap fraction from 0.4 to 6.3% at 2 m above ground level (Zhang et al., 2023; Zhang et al., 2022). Logging gaps are therefore an increasingly significant land cover, indicating ~1 Mha of forest has undergone canopy opening to this extreme degree across Borneo alone by 2015 as a direct result of logging. This has major implications for regional-scale ecosystem functioning due to the extent of selective logging across Southeast Asia (Stibig et al., 2014). As 70% of tropical forest is considered modified, mainly as a result of selective logging (Potapov et al., 2008), land cover change from intact closed canopy tropical forest to logging gaps is a process of increasing global importance.

While naturally occurring canopy gaps play an essential role in maintaining of forest structural complexity and biodiversity (Muscolo et al., 2014), more extensive logging gaps in tropical forest can have negative consequences, from increasing susceptibility to fire (Cochrane, 2001; Matricardi et al., 2013) to long-term impacts on vegetation population dynamics and diversity that can persist for over half a century post-disturbance (Xu et al., 2015; Yamada et al., 2013). Canopy gap creation can drastically alter local-scale soil physicochemical properties and microclimate (Denslow et al., 1998; Hartmann et al., 2012; Marthews et al., 2008; Ostertag, 1998; Saner et al., 2009; Scharenbroch and Bockheim, 2007, 2008b; Zhu et al., 2003; Zirlewagen and Von Wilpert, 2001) which may have knock-on effects for soil microbial communities and the vital biogeochemical processes they regulate. Despite this, the majority of studies of canopy gaps have focussed on aboveground communities and processes (see Muscolo et al., 2014), and few have evaluated belowground impacts on soil microbial community attributes related to ecosystem functioning (Li et al., 2019; Yang Y. et al., 2017).

Forest gaps can function as nutrient cycling “hotspots” (Schliemann and Bockheim, 2011), with studies observing increased rates of organic matter decomposition (Lin et al., 2015), soil nitrogen (N) mineralisation and nitrification (Denslow et al., 1998; Ritter, 2005), and increased bioavailability of limiting soil nutrients such as phosphorous (P) and N (Hu et al., 2016; Scharenbroch and Bockheim, 2008a). Increased rainfall reaching the topsoil has been found to cause erosion and leaching, reducing overall soil fertility in gaps (Arunachalam and Arunachalam, 2000), while soil compaction in landings and skid trails through use of heavy machinery for timber extraction is another major disturbance specifically associated with selective logging (Alexander, 2012; Grigal, 2000; Hartmann et al., 2013; Malmer and Grip, 1990; Marshall, 2000). Along with the removal of organic matter, these disturbances can have significant consequences for soil microbial communities and function (Hartmann et al., 2012).

Disturbance intensity and gap age can determine the extent and direction of change in soil properties (Denslow et al., 1998; Muscolo et al., 2007a; Ritter, 2005), but impacts on soil microbial communities are complex and not necessarily unidirectional. Attributes such as total microbial, bacterial, and fungal biomass have been found to be lower in gaps relative to intact forest due to reduced litter inputs and root densities (Arunachalam and Arunachalam, 2000; Schliemann and Bockheim, 2014). Other studies detected no differences (Luizão et al., 1998), or observed greater microbial biomass in small gaps (Muscolo et al., 2007a; Yang Y. et al., 2017) that may decrease again with increasing gap size (Muscolo et al., 2010; Muscolo et al., 2007a, 2007b; Yang Y. et al., 2017). Biomass of certain microbial groups may also be differentially affected, for example, bacteria favoured over fungi with increasing disturbance intensity (Arunachalam and Arunachalam, 2000), and microbial community structure, diversity, and activity may vary according to gap size along with soil conditions (Li et al., 2019; Xuan et al., 2018; Yang B. et al., 2017; Yang Y. et al., 2017).

A small number of recent studies have evaluated the effects of selective logging of tropical forest in Southeast Asia on soil microbial community structure, diversity (Kerfahi et al., 2014; Lee-Cruz et al., 2013; McGuire et al., 2015; Tripathi et al., 2016; Robinson et al., 2020), and function (Robinson et al., 2020). Following that dipterocarps are obligate ectomycorrhizal (EcM) associating trees (Taylor and Alexander, 2005; Brearley, 2012), fungal community attributes, particularly EcM fungal relative abundance and diversity, have been shown to be highly sensitive to logging disturbance (Kerfahi et al., 2014; McGuire et al., 2015). Bacteria and protists appear to be more resilient (Lee-Cruz et al., 2013; Tripathi et al., 2016) through a strong association with soil properties often independent of land-use type (Tripathi et al., 2012). However, these previous studies compared selectively logged and old-growth forest across large spatial scales using geographically separate study sites, potentially complicating relative site and disturbance effects, and without consideration of heterogeneity across selectively logged forest due to coarse spatial granularity of sampling. Few studies have investigated the impacts of logging on soil biogeochemical cycling in lowland dipterocarp forest, including total soil respiration (Adachi et al., 2006; Saner et al., 2009; Saner et al., 2012). Even fewer have attempted to quantify the effects on heterotrophic soil microbial respiration (Riutta et al., 2021), that is, the flux of carbon dioxide (CO_2_) from soil to the atmosphere resulting from microbial activity and decomposition of organic material, despite being a major component of the terrestrial C cycle and playing a crucial role in soil C sequestration, determining net accumulation or release of soil C (He et al., 2022; Tao et al., 2023).

Logging has the potential to greatly alter soil microbial communities and functions in tropical rainforests, but effects remain unresolved. To address this knowledge gap, we surveyed forest structure, soil microbial communities, soil properties, and nutrient and C cycling in co-located logging gaps and intact Bornean lowland dipterocarp rainforest to test the following hypotheses (see previous three paragraphs for corresponding references):

(1) Logging will significantly impact soil microbial biomass, community composition, and diversity, and these differences will relate to disturbance intensity (gap size and structure, i.e., canopy openness).
(2) Soil fungal communities will be more sensitive to logging than soil bacterial communities.
(3) Logging-induced alterations in soil microbial communities will correspond to variation in soil physicochemical properties and soil functioning (enzyme activity, nutrient supply rates, and microbial heterotrophic respiration).

## 2 Materials and methods

### 2.1 Study site

**Figure 1:**
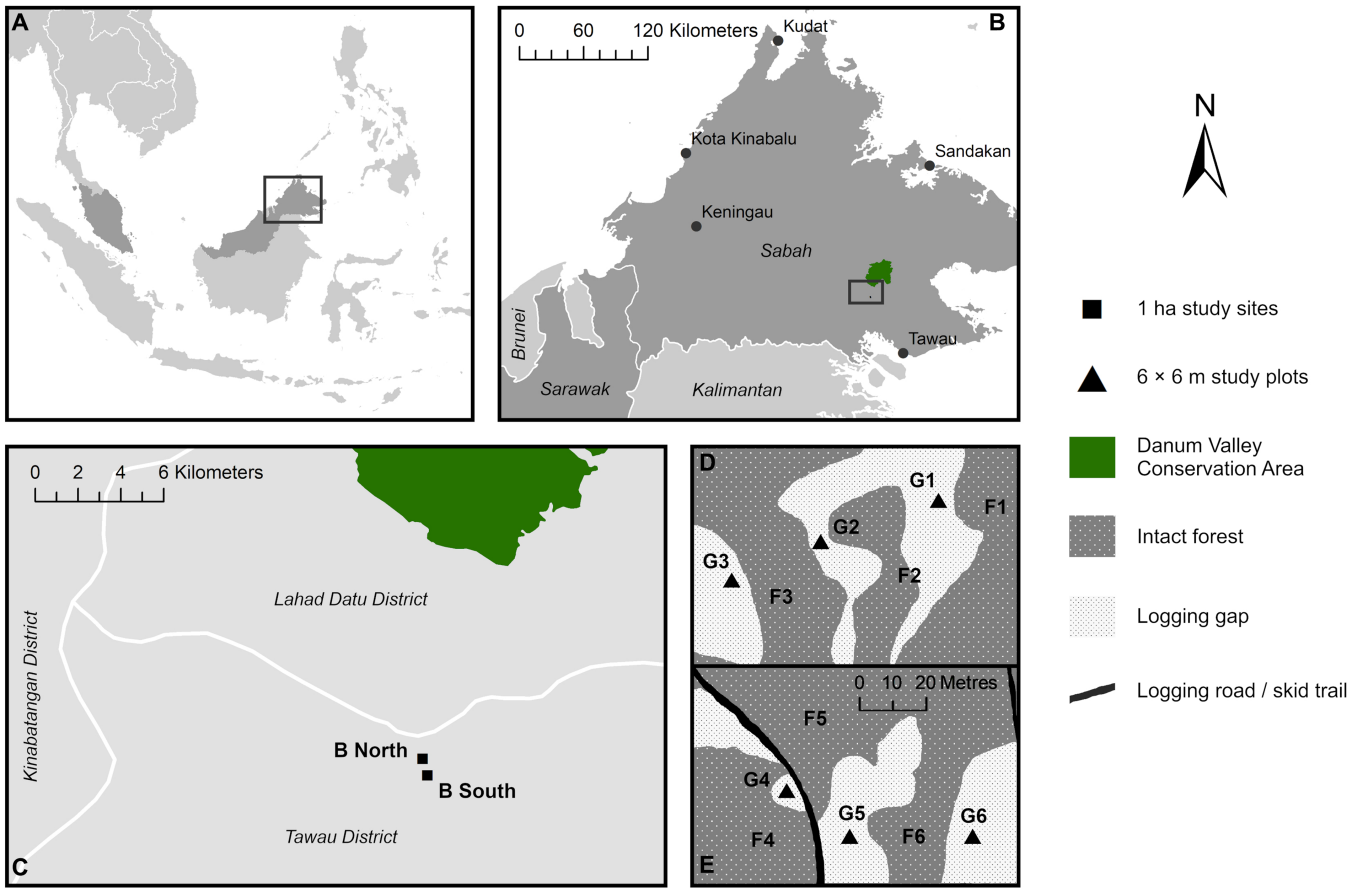
Map of sampling locations in northern Malaysian Borneo **(A)** in the state of Sabah **(B)**, within two 1 ha study sites situated in selectively logged forest in proximity to the Danum Valley Conservation Area old-growth forest reserve **(C)**. Approximate distribution of intact forest and logging gaps and the twelve 6 × 6 m study plots in the two study sites B South **(D)** and B North **(E)** based on field observation [panels **(D,E)** are presented at the same spatial scale].

This study was conducted in selectively logged lowland dipterocarp rainforest in the state of Sabah, northern Malaysian Borneo. The climate is characterised as moist tropical (average daily temperature 27°C, annual precipitation 2,600–2,700 mm) without distinct seasonality but may experience irregular inter-annual dry periods with an average total of ~1.4 months per year (Kumagai and Porporato, 2012; Walsh and Newbery, 1999). Sampling was carried out within two existing 1 ha research plots within the Kalabakan Forest Reserve (Figure 1; B South 4.732°N, 117.619°E and B North 4.739°N, 117.617°E; coded as SAF-01 and SAF-02, respectively, in the ForestPlots database), situated within the large-scale forest fragmentation study Stability of Altered Forest Ecosystems (SAFE) Project (Ewers et al., 2011). These plots (hereafter referred to as sites) were previously established for long-term evaluation of forest C cycling and productivity as part of the Global Ecosystem Monitoring (GEM) network (Malhi et al., 2015; Riutta et al., 2018). The two sites chosen for this study had similar land-use histories of heavy logging, both having been selectively logged four times: first in the mid-1970s (approximately 113 m^3^ ha^−1^ timber removed) followed by three rounds 1990–2008 (approximately 37–66 m^3^ ha^−1^ cumulative timber removed) (Fisher et al., 2011; Riutta et al., 2018).

### 2.2 Sampling design

Logging gaps in the two sites were not discrete but formed a mostly contiguous matrix with varying degrees of canopy openness. Twelve 6 × 6 m plots were distributed equally across both sites (Figures 1D,E). Three plots were established in each vegetation type per site as co-located contrasting pairs (<50 m between paired logging gap and intact forest plots), totalling six plots in logging gaps (G1-G6) and six in the intact forest (F1-F6) (Supplementary Figure 1). Plot size was chosen relative to the widths of logging gaps studied to avoid proximity to the intact forest edge. Widths of logging gaps at the narrowest point ranged from approximately 12–30 m, estimated from aerial images taken by drone survey (Cheerson CX-20 Auto-Pathfinder fitted with Apeman Action Camera). Each plot was subdivided into nine 2 × 2 m subplots, of which six were randomly chosen for sampling of soil microbial communities, physicochemical properties, nutrient supply rates, and environmental and forest structural characteristics, with the subplot centre marked as the sampling point.

### 2.3 Environmental and forest structural characteristics

Environmental and forest structural characteristics were measured upon soil sample collection in November 2016 unless otherwise stated. Location and elevation were recorded at the centre of each plot using GPS. The slope was measured at each sampling point using a clinometer. Data loggers equipped with soil moisture and temperature probes (Delta-T Devices Ltd., Cambridge, UK) were installed in the centre of each plot to record hourly microclimate measurements between November 2016 and March 2018 (490 days). Due to equipment failure during this period, only soil moisture data from 75 continuous days (November 2016–January 2017) and soil temperature data for 26 continuous days (November 2016–December 2016) in the BS site were used for consistency in statistical analysis (minimum of three data loggers per vegetation type in one site). Photosynthetically active radiation (PAR) was measured at each sampling point above understorey vegetation using a light meter (PP Systems, USA). Canopy openness was measured by taking hemispherical photographs at each sampling point using an Opteka 180° 6.5 mm fish-eye lens (Samyang Optics, Masan, South Korea) and a digital Canon 400D camera (Canon, Tokyo, Japan). The canopy gap fraction was calculated from images using the Hemisfer program version 2.2 (Swiss Federal Institute for Forest, Snow and Landscape Research). The diameter at breast height (DBH; 1.3 m from the ground) of all trees with DBH >3 cm was recorded in all plots.

### 2.4 Soil microbial community attributes and physicochemical properties

Three 10 cm depth soil cores were taken around each sampling point using a 3 cm diameter gouge auger for analysis of soil microbial community attributes and physicochemical properties in November 2016. Organic soil layer depth was measured before separation from underlying mineral soil and collection. Soil samples were bulked per sampling point (*n* = 6 composite samples per plot), sealed in Ziploc bags, and transported to a field laboratory. One additional soil sample was taken at three randomly selected sampling points per plot with a volumetric core (4 cm diameter, 8 cm depth) for determining soil bulk density and volumetric moisture content. Each composite sample was hand-homogenised, and subsamples were taken for analysis of soil microbial community attributes. These were frozen at −20°C on the day of collection and transported on ice to the UK. The remaining soil was transported to the Sabah Forest Research Centre, Sepilok for physicochemical analysis. Further samples were taken in March 2018 in the same way for soil enzyme analysis; samples were frozen on collection and transported on ice to the Smithsonian Tropical Research Institute, Panama. While seasonal differences between soil sampling rounds are unlikely (Section 2.1), parameters analysed on samples from the first (soil microbial communities and physicochemical properties) and second (soil enzymes and heterotrophic respiration) were not included together in any statistical test. Therefore, we do not expect differences in sampling dates to affect comparisons of soil characteristics between vegetation types or relationships with gap size.

Soil microbial communities were analysed by amplicon sequencing at the UK Centre for Ecology & Hydrology, Wallingford, using the same methodological pipeline described in Robinson et al. (2020) (see Supplementary Materials and Methods for detailed description). Total soil microbial biomass carbon (MBC) was analysed at Lancaster University. MBC was determined using a modified chloroform fumigation extraction method (Brookes et al., 1985; Vance et al., 1987; see Supplementary Materials and Methods for detailed description). Total C contents of extracts were measured using a Total Organic Carbon (TOC) Analyser (TOC-L, Shimadzu Corporation, Kyoto, Japan), and MBC was calculated as *μ*g g^−1^ dry soil.

Soil enzyme activity (maximum potential activity, *V*_max_) was determined on samples from four randomly selected sampling points per plot (a reduced number of samples were analysed for logistical reasons) and for nine enzymes involved in C, N, P, and S cycling: *α*-glucosidase and *β*-glucosidase (degradation of α- and β-bonds in glucose), cellobiohydrolase (degradation of cellulose), *β*-xylanase (degradation of hemicellulose), *N*-acetyl *β*-glucosaminidase (degradation of *N*-glycosidic bonds), phosphomonoesterase and phosphodiesterase (degradation of monoester- and diester-linked simple organic phosphates), and sulphatase (degradation of ester sulphates). Enzyme activity was determined using fluorometric assays with 100 *μ*M methylumbelliferone (MU)-linked substrates (Marx et al., 2001), with five replicate micro-plates for each soil sample and incubated at 25°C (representing soil temperature). Enzyme activities (*V*_max_) are expressed on the basis of activity at soil temperature per dry weight soil.

Soil pH was measured on fresh soils in deionised water (Landon, 1984). The remaining soil was air-dried and passed through a 2 mm sieve. Total soil C and N contents were determined by dry combustion at 900°C using an Elementar Vario Max CN analyser (Elementar Analysensysteme, Hanau, Germany). Soil total P was digested by sulphuric acid-hydrogen peroxide (Allen, 1989). Inorganic P was extracted using Bray No. One extractant (Bray and Kurtz, 1945). P contents of digests and extracts were determined using the molybdenum-blue method (Anderson and Ingram, 1993) and read at 880 nm on a spectrophotometer (HITACHI-UV–VIS, Japan). Soil texture (% sand, silt, and clay) was determined using the particle size distribution test (Day, 1965) on one bulked sample per plot (*n* = 12). Bulk density was determined after the removal of roots and stones by oven drying at 105°C for 48 h (Emmett et al., 2008) and averaged to one value per plot (*n* = 12).

### 2.5 Soil nutrient supply rates

Soil nutrient availability was measured using 10 cm^2^ resin ion exchange membranes (PRS^®^ Probes, Western AG, Saskatoon, Canada) for anions NO_3_^−^, P^−^, and S^−^, and cations NH_4_^+^, Ca^+^, Mg^+^, K^+^, Fe^+^, Mn^+^, Cu^+^, Zn^+^, B^+^, Pb^+^, Al^+^, and Cd^+^. Probes were installed during soil sampling in November 2016, arranged in four pairs (one cation and one anion probe) around each sampling point and inserted to a depth of 10 cm. Probes were removed from soil after 7 days, cleaned on collection with distilled water, and shipped to the manufacturer for chemical analysis. Probes were bulked per sampling point and eluted with 0.5 M HCl for 1 h. NO_3_^−^ and NH_4_^+^ concentrations were measured by colorimetric automated flow injection analysis. Concentrations of all other ions were measured using inductively coupled plasma-optical emission spectroscopy. Nutrient supply rates for each element are given as amounts per ion exchange membrane for the duration of burial (*μ*g probe^−1^ week^−1^).

### 2.6 Soil heterotrophic respiration

Soil cores were collected at three randomly chosen sampling points per plot for controlled laboratory incubations in March 2018 (a reduced number of sampling points were sampled for logistical reasons). Four soil cores were collected around each sampling point using plastic piping (8 cm deep, 4 cm internal diameter) and kept intact by sealing ends with rubber lids to preserve soil structural properties. Intact cores were used to retain soil physicochemical and structural properties influencing soil water and gas dynamics along the soil profile (e.g., Briones et al., 2014). Cores were stored in a cool box with frozen gel packs before transportation to Lancaster University, UK, where they were refrigerated at 4°C prior to incubation.

One core from each sampling point was used to determine soil water holding capacity (WHC). The remaining cores were installed in 1 L Mason jars in a randomised block design and incubated at a constant temperature of 24°C (average soil temperature across all intact forest and logging gap plots measured over the study period). Cores were adjusted to and maintained at 60 (±3) % soil WHC (representing the average measured across all plots) using a synthetic rain solution based on rain chemistry data from the nearby Danum Valley Field Station (4.95°N, 117.79°E) obtained from the World Data Centre for Precipitation Chemistry (Vet et al., 2014).^1^ Jars were sealed following an equilibration period of 1 week. CO_2_ fluxes were measured using a Picarro G2508 greenhouse gas analyser (Picarro Instruments, USA) and calculated as *μ*g CO_2_-C cm^−2^ h^−1^ (see Supplementary Materials and Methods for detailed description).

### 2.7 Molecular data pre-processing

Only ASVs assigned to the kingdoms of bacteria and fungi were retained for downstream analysis (99.34 and 99.73% of total 16S and ITS reads, respectively). Eight samples had abnormally low read counts in all libraries, including all samples plot G6 in B North. We therefore excluded these samples from all subsequent analyses. Singleton ASVs across all samples were removed (two fungal ASVs), and sample sequencing depth was normalised by rarefying to the minimum read counts of 13,498 (bacteria), 7,124 (total fungal), 1,983 (saprotrophic fungal), 22 (mycorrhizal fungal), 13 (EcM fungal), 501 (pathogenic fungal), and 19 (parasitic fungal). Fungal functional guild classifications were assigned to ASVs using the FUNGuild annotation tool (Nguyen et al., 2016). Only ASVs with unambiguous (non-multiple) classifications of “probable” or “highly probable” confidence rankings were considered for analysis. These were used for calculating the relative abundances of fungal guilds and sub-setting saprotrophic, mycorrhizal, EcM, pathogenic, and parasitic fungal datasets for analysis of diversity and community composition. Alpha diversity indices (ASV richness and Shannon index) and fungal guild relative abundances were calculated using rarefied datasets. All sequencing data pre-processing was conducted in R version 4.2.2 (R Core Team, 2018) using the phyloseq package (McMurdie and Holmes, 2013).

### 2.8 Statistical analyses

All statistical analyses were conducted in R version 4.2.2 or 4.4.0 (R Core Team, 2018), and significance was considered at the *p* < 0.05 level. Soil microbial community data were Hellinger-transformed prior to analysis (Legendre and Borcard, 2018) to control for the effect of rare taxa and merged at the plot level to control for spatial pseudoreplication. Soil microbial community compositions were visualised with PCoA using Bray–Curtis dissimilarities. Differences in soil microbial community composition between vegetation types were tested with PERMANOVA, and the homogeneity of multivariate dispersion was evaluated (Oksanen et al., 2019). The soil properties explaining the largest proportion of variance in soil microbial community dissimilarities were identified through backward selection with PERMANOVA, where variables with the highest *p*-values in marginal tests were sequentially removed until all predictors were significant. All permutational tests were run with 9,999 permutations and restricted by site to control for the nested sampling design using the permute R package (Simpson et al., 2019).

Differences between logging gaps and intact forest for environmental and forest structural characteristics, soil properties, and univariate soil microbial community attributes were tested using linear models (LMs) on plot-averaged data to control for spatial pseudoreplication. Plot averaging was chosen over a mixed model approach to improve the normality of model residuals while showing similar trends. The effect of vegetation type was tested after site factor to control for site effects. *R*^2^ values are given after removing variance associated with site for all comparisons between logging gaps and intact forest, apart from microclimate characteristics which were only measured in one site (BS). Soil properties explaining variance in the soil microbial community attributes that significantly differed by vegetation type were identified by multiple linear regressions through stepwise forward and backward variable selection using AIC as the criterion. Prior to analysis, highly correlated soil physicochemical variables (Pearson’s *r* > 0.6 averaged over both sites) were identified and removed as necessary.

Relationships between canopy openness and univariate soil microbial community attributes, soil physicochemical properties, and functioning within logging gaps were tested using linear mixed-effects models (LMMs) with Satterthwaite degrees of freedom approximation (Bates et al., 2015; Luke, 2017) and plot ID as a random intercept term. Normality of LM and LMM residuals was evaluated using Shapiro–Wilk tests, and variables were transformed where necessary to improve model fit.

## 3 Results

### 3.1 Soil microbial community attributes in logging gaps and intact forest

**Figure 2:**
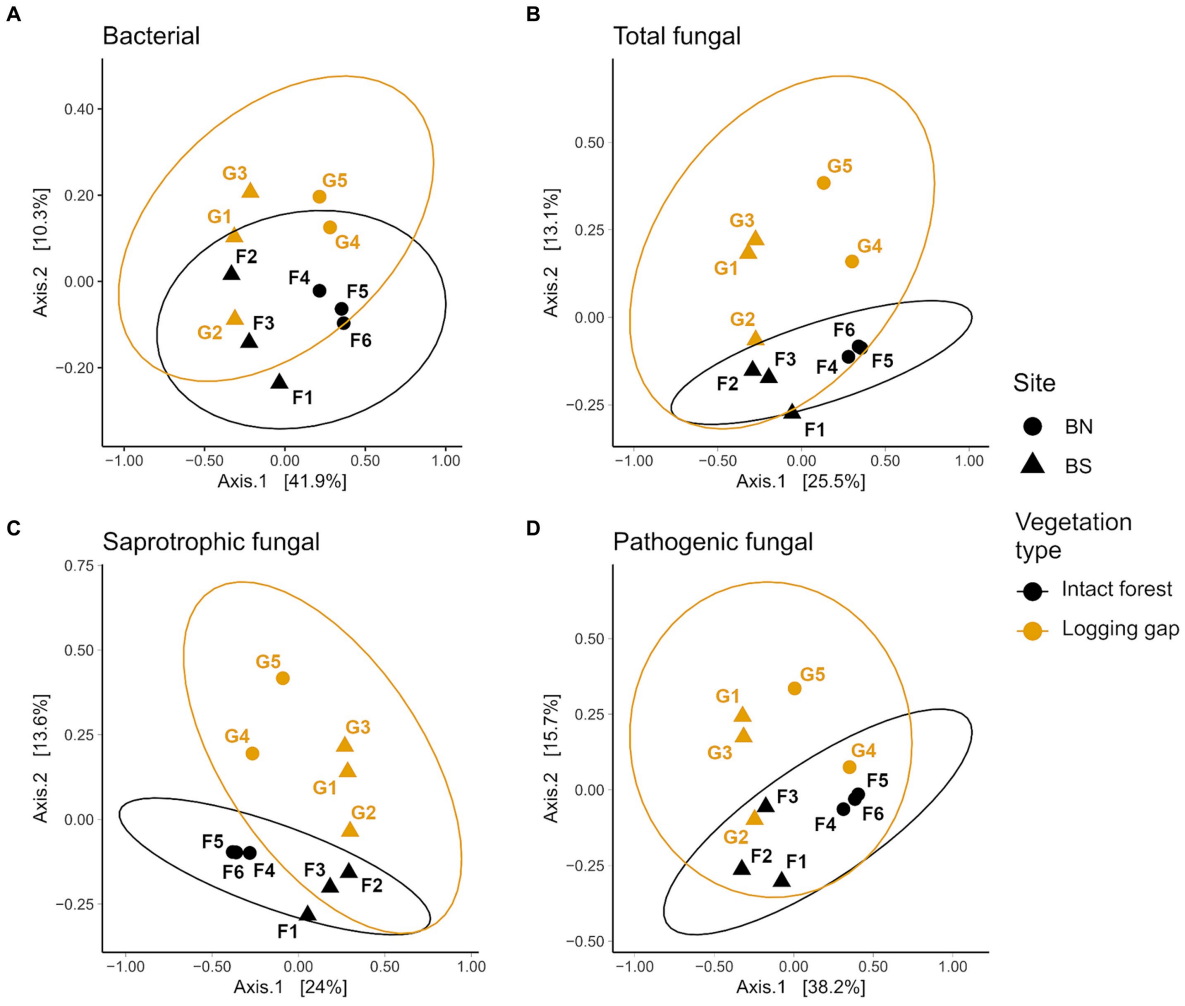
Principal coordinate analysis (PCoA) ordination of Bray–Curtis community dissimilarities for bacterial **(A)**, total fungal **(B)**, saprotrophic fungal **(C)** and pathogenic fungal **(D)** soil microbial groups that significantly differed (*p* < 0.05) between intact forest (black) and selective logging gaps (orange) identified with PERMANOVA after controlling for site, using data merged at the plot level (*n* = 11). Point labels denote plot ID, and shapes indicate locations of plots within the two sites B North (BN, circles) and B South (BS, triangles). Ellipses represent 95% confidence with t-distribution.

**Figure 3:**
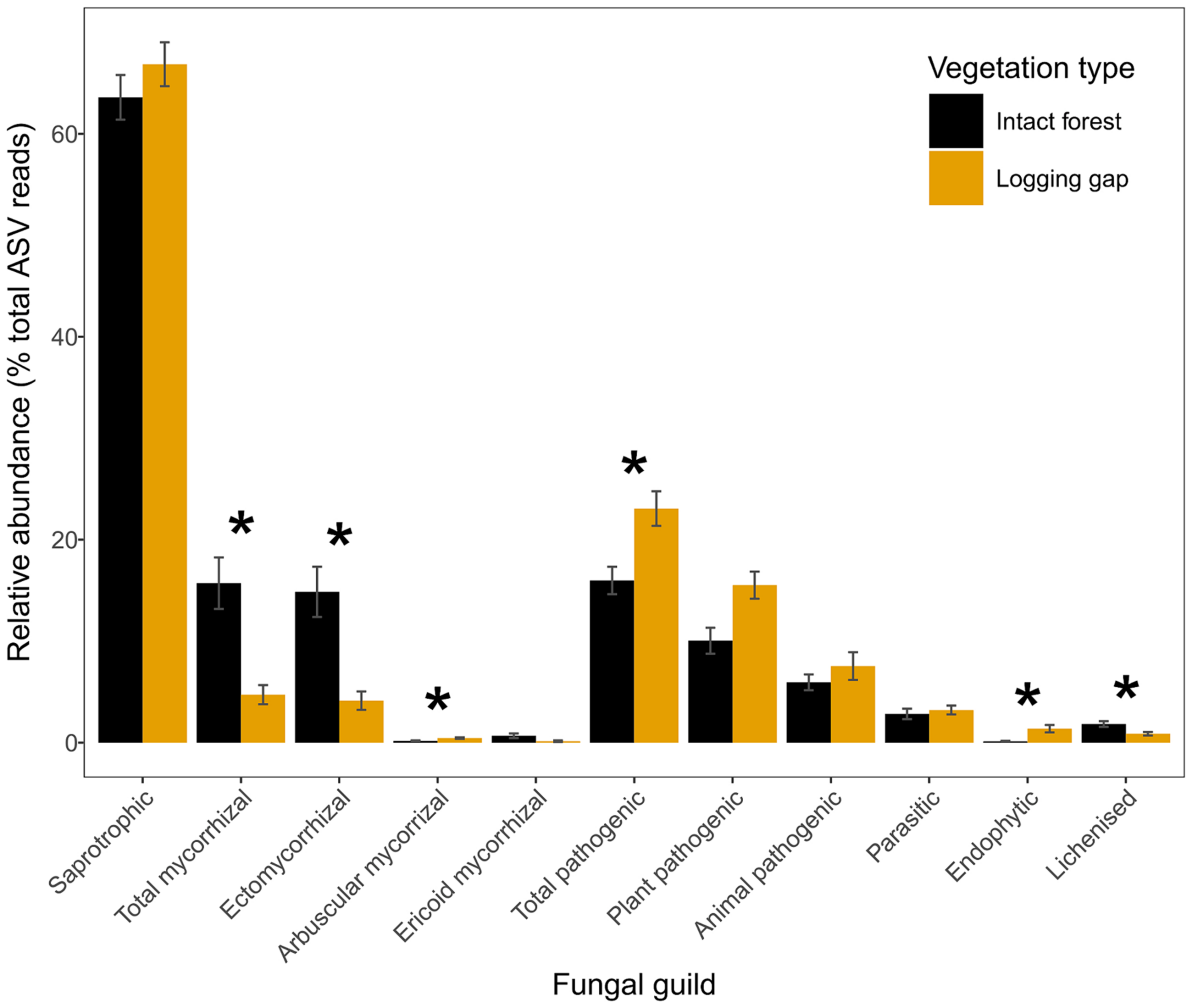
Relative abundances of fungal guilds for intact closed canopy forest and selective logging gaps. Error bars represent ±1 standard error. Asterisks indicate statistically different groups by vegetation type identified in ANOVA after controlling for site (*p* < 0.05). Mycorrhizal and pathogenic guilds have been further divided into subtypes as indicated.

A total of 10,913 bacterial ASVs (representing 40 phyla; 267 genera) and 13,848 fungal ASVs (12 phyla; 590 genera) were detected across all samples (Supplementary Figure 2). Bray–Curtis community dissimilarities significantly differed between logging gaps and intact forest for bacteria (*R*^2^ = 0.10, *F*_1,8_ = 1.01, *p* = 0.034; Figure 2A) and total fungal (*R*^2^ = 0.13, *F*_1,8_ = 1.34, *p* = 0.005), saprotrophic fungal (*R*^2^ = 0.14, *F*_1,8_ = 1.43, *p* = 0.005), and pathogenic fungal groups (*R*^2^ = 0.15, *F*_1,8_ = 1.60, *p* = 0.005) (Figures 2B–D). No significant difference between vegetation types was detected was detected for mycorrhizal, EcM, or parasitic fungal groups. Multivariate dispersion of community dissimilarities was homogenous between vegetation types for all soil microbial groups. Relative abundances of mycorrhizal, EcM, and lichenised fungi were significantly lower in logging gaps relative to intact forest, while relative abundances of AM, pathogenic, and endophytic fungi were significantly higher in logging gaps (Table 1 and Figure 3). No significant differences were detected between logging gaps and intact forest in MBC or alpha diversity indices (ASV richness and Shannon diversity index) for any soil microbial group.

**Table 1:** Means (±1 SD) of all soil microbial community attributes in intact closed canopy forest and selective logging gaps.

Parameter	Soil microbial group	Vegetation type		*R* ^2^	*F*	*p*
		Intact forest	Logging gap			
Total microbial biomass C (*μ*g g^−1^ dry soil)	–	321.3 ± 226.8	149.4 ± 56.5			
Richness (no. observed ASVs 10 reads^−1^)	Bacteria	0.42 ± 0.05	0.47 ± 0.04			
	Total fungi	0.70 ± 0.20	0.76 ± 0.13			
	Saprotrophic fungi	0.65 ± 0.22	0.67 ± 0.15			
	Mycorrhizal fungi	2.82 ± 0.22	2.72 ± 0.53			
	Ectomycorrhizal fungi	3.29 ± 0.63	2.62 ± 0.88			
	Pathogenic fungi	0.51 ± 0.16	0.52 ± 0.08			
	Parasitic fungi	1.26 ± 0.49	1.42 ± 0.25			
Shannon alpha diversity	Bacteria	4.98 ± 0.24	4.97 ± 0.23			
	Total fungi	4.34 ± 0.57	4.75 ± 0.30			
	Saprotrophic fungi	3.11 ± 0.67	3.33 ± 0.26			
	Mycorrhizal fungi	1.41 ± 0.13	1.36 ± 0.25			
	Ectomycorrhizal fungi	1.15 ± 0.24	0.92 ± 0.36			
	Pathogenic fungi	2.00 ± 0.50	2.24 ± 0.30			
	Parasitic fungi	0.44 ± 0.29	0.60 ± 0.23			
Fungal guild relative abundance (% total fungal ASV reads)	Saprotrophic fungi	63.60 ± 8.24	66.88 ± 5.51			
	Mycorrhizal fungi	15.71 ± 9.80	4.70 ± 3.00	0.48	7.28	0.027
	Ectomycorrhizal fungi	14.85 ± 9.43	4.11 ± 2.70	0.49	7.71	0.024
	Arbuscular mycorrhizal fungi	0.18 ± 0.12	0.45 ± 0.12	0.65	14.64	0.005
	Ericoid mycorrhizal fungi	0.68 ± 0.82	0.14 ± 0.24			
	Orchid mycorrhizal fungi	0.000 ± 0.000	0.002 ± 0.005			
	Pathogenic fungi	15.98 ± 4.35	22.98 ± 3.82	0.46	6.86	0.031
	Plant pathogenic fungi	10.05 ± 5.27	15.66 ± 3.18			
	Animal pathogenic fungi	5.94 ± 2.47	7.32 ± 4.48			
	Parasitic fungi	2.83 ± 1.67	3.27 ± 1.32			
	Endophytic fungi	0.14 ± 0.06	1.42 ± 1.40	0.77	26.30	0.001
	Lichenised fungi	1.83 ± 0.94	0.85 ± 0.49	0.58	11.18	0.010

SDs were calculated using plot-averaged values to match statistical tests. Statistics are given for attributes that significantly differed between vegetation types as identified with ANOVA after controlling for site (*p* < 0.05).

### 3.2 Environmental and soil characteristics and soil functioning in logging gaps and intact forest

Plot average canopy gap fractions varied between 6.41 –10.8% in intact forest and 27.6–63.4% in logging gaps (Supplementary Figure 3A). Basal area in intact forest varied between 8.26–50.93 m^2^ ha^−1^ and was significantly greater than logging gaps where basal area was zero in all plots except G4 (21.30 m^2^ ha^−1^) (Supplementary Figure 3B and Table 2). No significant differences were found between vegetation types for most of the tested environmental and soil properties, but logging gaps were associated with significantly higher PAR, maximum soil moisture content, soil pH (Supplementary Figure 3C), and soil bulk density (Supplementary Figure 3D) and lower soil inorganic P content (Supplementary Figure 3E and Table 2). Soil silt content was marginally significantly higher in logging gaps relative to intact forest (*R*^2^ = 0.40, *F*_1,8_ = 5.23, *p* = 0.051). Soil NO_3_^−^ supply rates were significantly reduced in logging gaps (Table 2), while soil heterotrophic respiration rates were marginally significantly lower in logging gaps (*R*^2^ = 0.39, *F*_1,8_ = 5.04, *p* = 0.055). No significant differences were found between logging gaps and intact forest in the activity of any enzymes assayed.

**Table 2:** Means (±1 SD) of all environmental and forest structural characteristics, soil properties, and measures of soil functioning in intact closed canopy forest and selective logging gaps.

Group	Parameter	Vegetation type		*R* ^2^	*F*	*p*
		Intact forest	Logging gap			
Topography	Altitude (m)	481.00 ± 111.52	459.40 ± 116.56			
	Slope (°)	17.94 ± 7.27	11.27 ± 5.40			
Microclimate	Mean soil temperature (°C)	26.41 ± 3.38	27.26 ± 3.65			
	Max. soil temperature (°C)	27.60 ± 3.47	31.80 ± 5.62			
	Min. soil temperature (°C)	25.37 ± 3.15	24.87 ± 3.51			
	Mean soil moisture (%)	38.24 ± 5.95	39.77 ± 8.80			
	Max. soil moisture (%)	48.23 ± 1.54	62.33 ± 2.15	0.95	76.66	0.001
	Min. soil moisture (%)	33.00 ± 7.40	33.70 ± 5.74			
	PAR (*μ*mol m^−2^ s^−1^)	6.88 ± 10.14	94.57 ± 65.16	0.67	9.70	0.014
Forest structural	Canopy openness (%)	8.77 ± 1.54	50.60 ± 14.26	0.93	114.06	<0.001
	Basal area (m^2^ ha^−1^)	31.12 ± 16.77	4.26 ± 9.53	0.58	11.21	0.010
Soil physicochemical	pH	4.66 ± 0.54	5.25 ± 0.44	0.69	17.73	0.003
	Organic layer depth (cm)	4.06 ± 0.78	3.88 ± 2.45			
	Sand (%)	61.67 ± 6.41	58.40 ± 8.17			
	Silt (%)	13.50 ± 2.43	17.20 ± 4.09			
	Clay (%)	24.83 ± 4.96	24.20 ± 5.12			
	Bulk density (g cm^−3^)	0.87 ± 0.13	1.06 ± 0.09	0.49	7.80	0.023
	C (%)	3.85 ± 0.94	3.32 ± 0.64			
	N (%)	0.31 ± 0.09	0.28 ± 0.06			
	C: N ratio	12.60 ± 1.96	12.18 ± 1.39			
	Total P (*μ*g g^−1^)	230.07 ± 83.24	253.71 ± 103.59			
	Inorganic P (*μ*g g^−1^)	6.45 ± 1.68	4.50 ± 0.54	0.63	13.52	0.006
Soil nutrient supply rates (*μ*g probe^−1^ week^−1^)	NO_3_^−^	90.63 ± 76.96	49.08 ± 44.09	0.53	8.93	0.017
	P^−^	1.22 ± 0.45	1.50 ± 1.15			
	S^−^	21.59 ± 12.07	30.49 ± 8.37			
	NH_4_^+^	20.20 ± 9.08	12.95 ± 8.30			
	Ca^+^	327.00 ± 270.55	481.58 ± 314.45			
	Mg^+^	129.48 ± 70.28	177.43 ± 93.99			
	K^+^	222.43 ± 67.45	214.13 ± 55.78			
	Fe^+^	6.44 ± 2.32	14.05 ± 13.33			
	Mn^+^	9.95 ± 2.85	12.24 ± 15.85			
	Zn^+^	0.73 ± 0.11	0.59 ± 0.23			
	Al^+^	10.96 ± 2.94	11.82 ± 3.96			
Heterotrophic soil respiration	CO_2_ efflux rate (*μ*g CO_2_-C cm^−2^ h^−1^)	71.30 ± 13.31	51.68 ± 14.63			
Soil enzyme activity (nmol g dry soil^−1^ min^−1^)	Phosphomonoesterase	45.08 ± 18.22	40.71 ± 18.23			
	Phosphodiesterase	8.16 ± 4.78	7.84 ± 6.31			
	*N*-acetyl-*β*-glucosaminidase	3.25 ± 1.07	3.13 ± 0.68			
	*β*-glucosidase	4.24 ± 1.56	5.14 ± 1.31			
	Sulphatase	3.21 ± 2.26	4.22 ± 2.38			
	*α*-glucosidase	0.35 ± 0.10	0.28 ± 0.07			
	*β*-Xylanase	1.76 ± 0.63	1.30 ± 0.20			
	Cellobiohydrolase	1.04 ± 0.45	1.15 ± 0.39			

SDs were calculated using plot-averaged values to match statistical tests. Statistics are given for attributes that significantly differed between vegetation types as identified with ANOVA after controlling for site (*p* < 0.05).

### 3.3 Relationships between soil microbial community attributes and soil properties

Bray–Curtis community dissimilarities for bacteria, total fungi, and pathogenic fungi were best explained by soil pH alone, while for saprotrophic fungi the selected predictors were pH and NO_3_^−^ supply rates (Table 3). Soil pH explained the largest proportion of variance in relative abundances of mycorrhizal (42.7%), EcM (45.1%), AM (68.4%), pathogenic (46.6%), endophytic (51.8%), and lichenised fungi (61.1%) (Supplementary Table 1). Although saprotrophic fungal relative abundance was not significantly affected by vegetation type, multiple regression analysis was also conducted as most fungal ASV reads were attributed to saprotrophic fungi (66.7% overall: 69.4% in logging gaps; 63.4% in intact forest) (Figure 3). Total soil P explained most variation in saprotrophic relative abundance (80.2%) after removing variance associated with site (Supplementary Table 1).

**Table 3:** PERMANOVA results of soil characteristics best explaining variation in soil microbial Bray–Curtis community dissimilarities, identified through backward selection using *p*-values in marginal tests controlling for site.

Soil microbial group	Predictor	*df*	Partial *R*^2^	*F*	*p*	Model *R*^2^
Bacteria	pH	1		5.23	0.014	0.37
	Error	9				
Total fungi	pH	1		2.81	0.002	0.24
	Error	9				
Saprotrophic fungi	pH	1	0.23	2.79	<0.001	0.34
	NO_3_^−^ supply rate	1	0.11	1.35	0.029	
	Error	8				
Pathogenic fungi	pH	1		4.76	0.007	0.35
	Error	9				

Variables are presented in the order entered in the model. Partial *R*^2^ represents the relative proportion of the explained variance of each predictor.

### 3.4 Relationships between canopy openness and soil microbial community attributes, soil properties, and soil functioning

**Figure 4:**
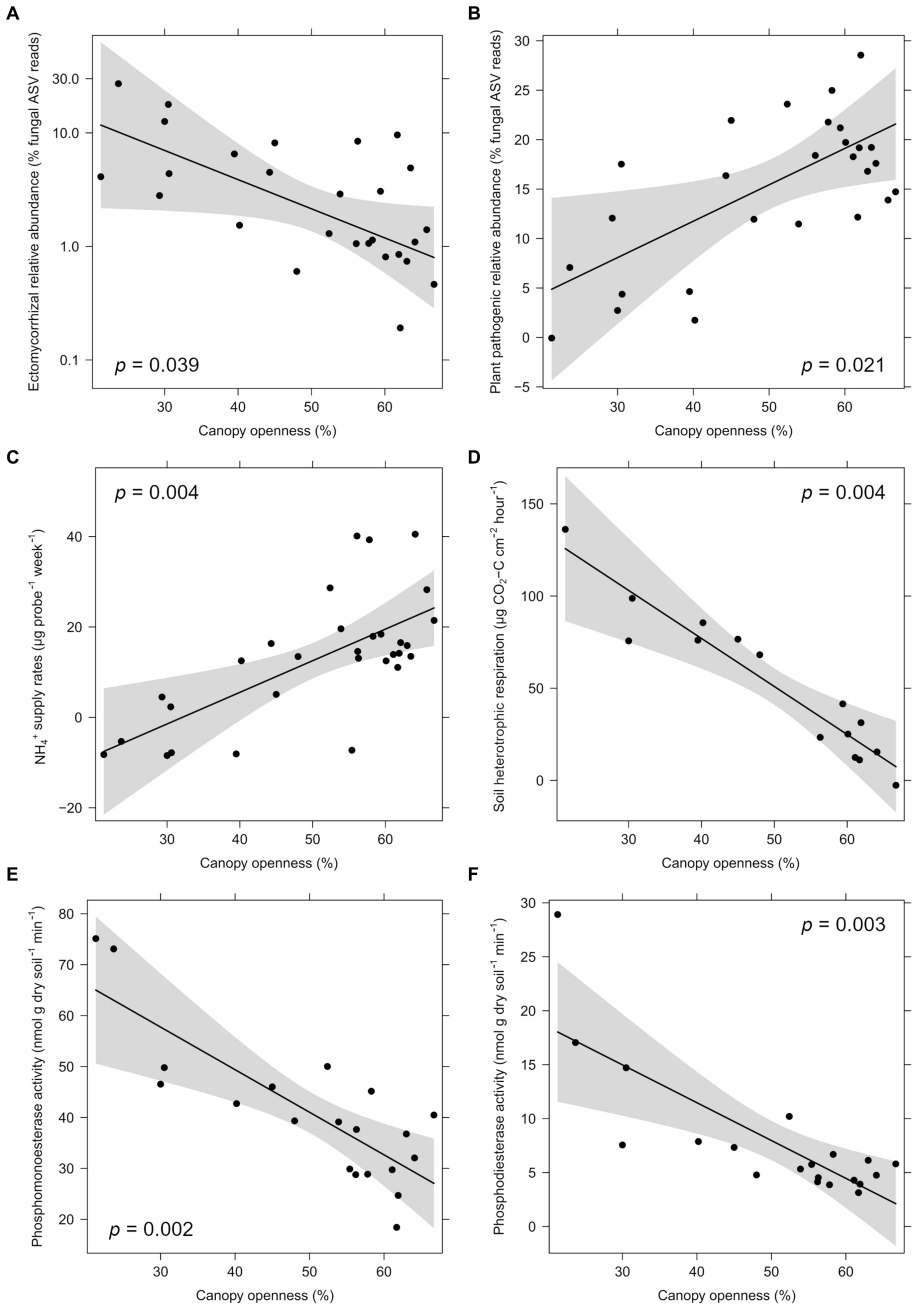
Partial regressions of significant effects of logging intensity (canopy openness) within gaps on fungal guild relative abundances **(A,B)**, nutrient supply rates **(C)**, soil heterotrophic respiration **(D)**, and enzyme activity **(E,F)**. Relationships and *p*-values are shown after controlling for site (fixed effect) and plot (random effect) using linear mixed models. Ectomycorrhizal relative abundance is presented on a log scale corresponding to statistical tests.

Within logging gaps, canopy openness was significantly negatively related to the relative abundance of total mycorrhizal and EcM fungi (Figure 4A) and significantly positively related to the relative abundance of plant pathogenic fungi (Figure 4B and Table 4). Disturbance intensity was not significantly related to community dissimilarities or alpha diversity indices of any soil microbial group. Canopy openness was significantly positively related to supply rates of NH_4_^+^ (Figure 4C), K^+^, and Zn^+^ and significantly negatively related to soil heterotrophic respiration (Figure 4D) and activity of enzymes phosphomonoesterase and phosphodiesterase (Figures 4E,F and Table 4).

**Table 4:** LMM results of significant relationships (*p* < 0.05) between logging disturbance intensity (canopy openness) and soil microbial attributes, soil physicochemical properties, and functioning within logging gaps.

Group	*Parameter*	*F*	*p*	*Direction*
Fungal guild relative abundance	Total mycorrhizal	5.81	0.024	−
	Ectomycorrhizal	4.73	0.039	−
	Plant pathogenic	6.08	0.021	+
Soil nutrient supply rates	NH_4_^+^	9.81	0.004	+
	K^+^	9.06	0.020	+
	Zn^+^	6.11	0.047	+
Soil heterotrophic respiration	Soil CO_2_ efflux rate	17.98	0.004	−
Soil enzyme activity	Phosphomonoesterase	13.68	0.002	−
	Phosphodiesterase	12.01	0.003	−

+ and − symbols denote positive and negative relationships, respectively.

## 4 Discussion

We found many soil microbial community attributes (total biomass and alpha diversity metrics) to be resistant to logging disturbance. Soil physicochemical properties and functions were also largely unaffected. However, we identified significant effects of logging on both bacterial and fungal microbial community composition and relative abundances of key soil fungal groups which corresponded with changes in certain soil physicochemical properties and a downregulation of some soil biogeochemical cycling processes. Through our use of co-located logged and intact forest plots (<50 m separation) to ensure the capture of spatial heterogeneity across selectively logged forest, we demonstrate the sensitivity of soil microbial communities and function to logging not previously shown.

The alteration of bacterial community composition with logging (Figure 2A) contrasts with previous observations in Bornean lowland dipterocarp rainforest that suggested bacterial community assemblages are broadly resilient to forest modification (Lee-Cruz et al., 2013; Tripathi et al., 2016). These previous studies compared old-growth and selectively logged forest using geographically separate study sites (kilometre-scale separation) where differences may be masked by site effects, as well as high spatial variation across selectively logged forest. Elsewhere in the humid tropics, studies have found soil bacterial community shifts with forest disturbance resulting from vegetation-mediated alterations in soil properties. As reviewed by Franco et al. (2019), Amazonian rainforest affected by slash-and-burn practices harbour distinct soil bacterial communities relative to primary forest (Cenciani et al., 2009; Mendes et al., 2015a, 2015b; Navarrete et al., 2015), while bacterial community composition may vary with land-use intensification in regenerating secondary forest after agricultural or pasture abandonment (de Carvalho et al., 2016). Disturbance impacts on bacterial community composition may be mediated by changes in soil properties, particularly as community dissimilarities are closely linked with soil pH (de Carvalho et al., 2016; Mendes et al., 2015a). Variation in bacterial metabolic functional characteristics along with community structure post-disturbance suggests taxonomic-functional adaptation of soil bacterial communities after forest clearance (Navarrete et al., 2015). In Costa Rican Atlantic Forest affected by hurricane, differences in bacterial community composition were attributed to the “taxonomic switching” of functional genera in response to altered canopy material soil inputs via canopy opening and increasing forest floor woody debris (Eaton et al., 2020). The effect of logging on bacterial communities observed here may arise from the regeneration status of trees within the logging gaps studied. Succession of soil microbial communities is inherently linked to that of plant communities (van der Heijden et al., 2008), and soil bacterial communities have been shown to recover rapidly after disturbance during forest reestablishment, followed by fast-growing pathogenic then slower-growing beneficial fungal communities (Song and Liu, 2018; Zhang et al., 2020), reflecting broader patterns observed in abandoned agricultural systems (Hannula et al., 2017). The absence of trees (DBH >3 cm) in all but one of the logging gap plots indicates these logging gaps are largely undergoing initial stages of secondary succession, where bacterial communities may be yet to reach a similar composition to that of climax communities.

Following the above successional process, fungal communities were also affected by logging, as shown by differences in total, saprotrophic and pathogenic fungal community dissimilarities between logging gaps and intact forest (Figures 2B–D), a reduction in the abundance of EcM and increase in AM and pathogenic fungi (Figure 3 and Table 1). These findings reflect the sensitivity of fungal communities to logging observed at larger spatial scales in lowland dipterocarp rainforest (Kerfahi et al., 2014; McGuire et al., 2015) and correspond with limited studies in other humid tropical forest systems including French Guiana (García de León et al., 2018), Costa Rica (Eaton et al., 2020), and Southwest China (Shi et al., 2016). The changes in the relative abundances of mycorrhizal groups reflect differences found in actively foraging fungal communities between old-growth and selectively logged forests in the same area (Robinson et al., 2020). This is likely a direct result of the removal of individuals of the Dipterocarpaceae, a family of EcM-associating trees (Brearley, 2012; Taylor and Alexander, 2005). Although dipterocarp species are targeted in selective logging for their economic value (Appanah and Turnbull, 1998), some individuals may remain even after repeated heavy logging as indicated by recent tree community survey of the 1 ha plots used in the present study (dipterocarp basal area in B North: 0.57 m^2^ ha^−1^; B South: 0.41 m^2^ ha^−1^) (Both et al., 2019; Robinson et al., 2020). Increased AM fungal relative abundance in logging gaps reflects a shift to non-dipterocarp, AM-associating vegetation, the dominant mycorrhizal type across tropical ecosystems (McGuire et al., 2008). These results also highlight the importance of remaining dipterocarp trees in maintaining mycorrhizal community composition after selective logging. Shifts from EcM- to AM-dominated communities may have important implications for biogeochemical cycling. The C sink from plants to EcM fungal mycelia has been estimated to be twice that of AM fungal mycelia (Hawkins et al., 2023). Indeed, a large allocation of photosynthetic C to EcM fungi is consistent with the findings of a girdling experiment in tropical Bornean forest that resulted in high mortality for dipterocarp species, which was correlated to the magnitude of the decline in soil CO_2_ emissions (Nottingham et al., 2022). EcM fungi have been further associated with soil C accumulation by competing with saprotrophs for nutrients (N and P) required for the breakdown of organic matter (Averill et al., 2014; Liu et al., 2018). A reduced EcM dominance therefore has potentially detrimental consequences for C storage in tropical forest soils. No differences were detected in mycorrhizal or EcM community composition between vegetation types, although care must be taken due to the small number of ASV reads used for assessing the community composition of these fungal groups which may limit sensitivity in detecting differences (see Section 2.7). While total mycorrhizal abundance may be reduced by alterations in vegetation community with logging, community structure may be more resilient to disturbance. The extramatrical mycelium produced by EcM fungi, shown to be largely retained after selective logging (Robinson et al., 2020), may extend into gaps from surrounding intact forest and could also provide an important inoculum for dipterocarp establishment during forest regeneration. Due to the early successional status of most of the logging gaps studied, we expect the similarity in EcM community composition between vegetation types is due to the influence of adjacent intact forest rather than the regeneration of EcM-associating trees within logging gaps.

The changes that were observed in soil microbial community attributes were accompanied by alterations in certain soil physicochemical properties. Soil pH was significantly higher in logging gaps relative to intact forest (Table 2 and Supplementary Figure 3C), despite higher soil moisture maxima which may be expected to result in the leaching of soil bases with an acidifying effect (Arunachalam and Arunachalam, 2000). This difference can therefore be attributed to the influence of vegetation rather than microclimate. Lower pH under intact forest is potentially a result of alterations in the quantity and quality of litter inputs. In regenerating secondary tropical forest, lower soil pH has been found in more mature stands (Robinson et al., 2015) as the breakdown of litter from older forest may release more humic acids into the soil (Melvin et al., 2011), while the amount of litterfall is higher under a closed canopy relative to gaps (Lin et al., 2015; Saner et al., 2009). Root exudation under intact forest may also contribute to rhizosphere acidification, for example, via the increased exudation of carboxylates that have been associated with P-limited systems (Lambers et al., 2006; Weemstra et al., 2016). Differences in soil pH would not be expected to result from logging residues left over in gaps as the decomposition of coarse woody debris has been observed to have an acidifying effect on soil (Spears and Lajtha, 2004; Khan et al., 2022). Soil bulk density was significantly higher in logging gaps relative to intact forest (Table 2 and Supplementary Figure 3D), presumably attributable to soil compaction by heavy machinery used for timber extraction (Alexander, 2012; Grigal, 2000; Hartmann et al., 2013; Malmer and Grip, 1990; Marshall, 2000) and reduced root infiltration (Saner et al., 2009). The linkages we observed between pH and soil microbial community assemblages are well established in temperate (Dupont et al., 2016; Lauber et al., 2009; Lauber et al., 2008; Rousk et al., 2010) and tropical ecosystems (de Carvalho et al., 2016; Jesus et al., 2009; Mendes et al., 2015a), including Southeast Asian forest where pH has been identified as the primary determinant of bacterial community structure and diversity (Tripathi et al., 2012). However, although increased soil pH levels associated with certain land-use practices in the tropics (e.g., agricultural crop and pastureland) have been shown to increase bacterial diversity as a result of these mechanisms (Tripathi et al., 2012), bacterial diversity was not found to be significantly higher in logging gaps relative to intact forest despite elevated pH levels towards neutral, suggesting microbial successional processes may instead act as a primary control on community attributes.

Most soil physicochemical properties and soil nutrient supply rates, and activity of all soil enzymes assayed did not differ between logging gaps and intact forest, apart from a significant reduction in inorganic P pools and NO_3_^−^ supply rates in gaps which may indicate some downregulation of P cycling and nitrification (Supplementary Figures 3E,F). This is contrary to many studies evaluating biogeochemical cycling rates in temperate forest gaps which observed increased nutrient availability, largely resulting from increases in soil temperature enhancing soil microbial activity and decomposition (Muscolo et al., 2014; Ritter, 2005; Scharenbroch and Bockheim, 2008a). However, nitrification can depend on disturbance intensity and gap age, with high rates in newly formed gaps (Ritter, 2005). The present study area has undergone multiple rounds of logging since the mid-1970s, with the youngest potential gap age at sampling being 8 years. Differences observed in NO_3_^−^ supply rates may indicate longer-term impacts of gap creation in heavily degraded tropical forests on nutrient cycling, which may be restricted due to decreased litter inputs (Lin et al., 2015; Saner et al., 2009), although analysis of the influence of gap age on nutrient dynamics and other soil properties was not possible in this study. Contrasts between our observations and those of other studies may also arise from system-specific impacts of logging on vegetation and associated plant litter and root chemistry, which are known to influence soil microbial communities and soil nutrient cycling (e.g., Leppert et al., 2017; Osborne et al., 2021; Song et al., 2023).

Mean soil heterotrophic respiration was lower in logging gaps relative to intact forest, although this difference was marginally non-significant (*p* = 0.055) possibly due to the limited number of samples used in laboratory incubation and high heterogeneity found across all plots (Table 2 and Supplementary Figure 3G). The significant negative relationship found between soil CO_2_ efflux and disturbance intensity (i.e., canopy openness) within gaps (Figure 4D) provides some evidence that logging downregulates soil microbial C cycling in tropical dipterocarp forest. Reduced heterotrophic soil respiration has been observed following clearance of dry deciduous tropical forest in India measured by laboratory assay (Sahani and Behera, 2001) and after harvest of Eucalyptus plantation in Congo inferred from field measurements (Epron et al., 2006). Under field conditions, it is likely that logging gaps would have a large effect on autotrophic respiration by altering root and rhizosphere communities. For example, in Malaysian dipterocarp forest, a previous study in Sabah found *in situ* soil CO_2_ efflux to be lower in logging gaps (Saner et al., 2009), a result consistent with a girdling experiment in dipterocarp forest, which found large declines in soil CO_2_ efflux associated with dipterocarp mortality (Nottingham et al., 2022). Saner et al. (2009) attributed the reduction of soil CO_2_ efflux in gaps mainly to shifts in vegetation characteristics, with lower fine-root biomass in gaps reducing the contribution of autotrophic plant root respiration to overall soil CO_2_ production, an effect likely exacerbated due to loss of EcM following dipterocarp removal (McGuire et al., 2015; Kerfahi et al., 2014) and an associated decline in autotrophic respiration (Nottingham et al., 2022). Riutta et al. (2021) found heterotrophic soil organic matter (SOM) respiration (i.e., excluding roots and mycorrhizae) to be greater in logged relative to old-growth dipterocarp forest through field manipulations. In logged forest, heterotrophic SOM respiration was found to be greater than total organic C soil inputs which indicates a net loss of SOM-C over time and would presumably result in a reduction in soil heterotrophic respiration in the long term. Owing to the potential advanced age of the logging gaps in this study, coupled with the slow recovery of tree communities and our targeted high spatial resolution sampling approach, our results may indicate hotspots of reduced soil microbial heterotrophic respiration in logging gaps that have not before been detected in Bornean tropical forest. This likely results from alterations in plant-C inputs (substrate availability) to the soil via reduced belowground C allocation from roots to the rhizosphere or by altered chemistry of plant litter inputs affecting microbial C-cycling processes (e.g., Bourget et al., 2023). While we recognise that some fine-root decomposition may contribute to overall higher soil heterotrophic respiration in cores with lower canopy openness, our findings indicate for the first time the importance of logging in suppressing soil microbial C cycling under controlled temperature and soil moisture conditions, which may reflect overall reduced rather than increased soil C accumulation by soil microbes. In temperate biomes, soil microbial heterotrophic respiration has been linked to severity of soil compaction associated with timber extraction, reducing microbial decomposition under more anaerobic conditions, increased physical protection of soil organic matter, and reduced gas diffusivity (Hartmann et al., 2013). We found greater soil bulk densities in logging gaps relative to intact forest, suggesting soil compaction may also contribute to reduced soil microbial heterotrophic respiration.

We found intensity of logging disturbance (i.e., canopy openness within gaps) corresponded to some fungal community attributes, soil enzyme activities, and N-cycling processes (Table 4). Canopy openness was significantly negatively related to total mycorrhizal and EcM relative abundances, and activity of soil phosphomonoesterase and positively associated with supply rates of certain key soil nutrients (NH_4_^+^, Mg^+^, K^+^, and Zn^+^) (Figures 4A,C). Increasing NH_4_^+^ supply rates with canopy openness may contradict assumptions about disturbance intensity and N cycling (Muscolo et al., 2007a). A positive relationship between gap size and N mineralisation rate has been observed in temperate (Ritter, 2005) and tropical (Denslow et al., 1998) systems, but only in newly created gaps (<17 months and 12 months old, respectively) where increased nutrient cycling rates have been attributed to decomposition of fresh litter inputs resulting from disturbance. The time since logging in the current study and the lack of difference in NH_4_^+^ supply rates between intact forest and logging gaps (Table 2) suggests a different mechanism underlying changes in N mineralisation. Mycorrhizae, particularly EcM, are now known to be able to mobilise soil N from organic sources and not be reliant on previous breakdown by other soil saprotrophs (Lindahl and Tunlid, 2015). A reduction in mycorrhizal relative abundance may subsequently increase the availability of organic N to be mineralised by other soil microorganisms (Averill et al., 2014). Similarly, the synthesis of phosphatase enzymes is associated with soil microbial mineralisation of organic P and usually increases with P limitation in tropical forest (Turner et al., 2018; Turner and Engelbrecht, 2011). Soil P cycling is heavily influenced by fungal community abundance, activity, and composition, with the relative dominance of either AM or EcM fungi strongly influencing P cycling pathways (Rosling et al., 2016). The corresponding decrease in EcM relative abundance and activity of soil phosphatase may indicate a functional shift in fungal communities and downregulation of soil P mineralisation with increasing disturbance intensity.

## 5 Conclusion

In conclusion, we found many soil microbial community attributes, soil properties, and functions to be resistant to selective logging, but through our use of co-located logged and intact study plots capturing spatial variation across selectively logged forest, we demonstrate logging significantly impacts the composition and abundance of key soil microbial groups (Hypothesis 1) including bacterial communities not before shown (Hypothesis 2), corresponding to alterations in certain soil properties and biogeochemical cycling processes in tropical forest (Hypothesis 3). A strong reduction in relative abundances of mycorrhizal and EcM fungi and a significant increase in AM fungi in logging gaps may have consequences for soil C dynamics (i.e., soil C storage), while the potential importance of EcM-associating dipterocarp trees remaining after selective logging is highlighted. Differences in all soil microbial community attributes corresponded to changes in soil pH. Within gaps, logging intensity was significantly positively related to supply rates of certain key soil nutrients, including a strong association with NH_4_^+^, and negatively related to mycorrhizal and EcM relative abundance, and phosphomonoesterase activity. Our findings suggest that mineralisation rates in logging gaps may be enhanced by reduced competition for organic soil N sources between EcM fungi and other soil saprotrophs, while P cycling pathways may be altered by functional shifts in fungal communities. A strong negative relationship between logging disturbance intensity and soil microbial heterotrophic respiration in logging gaps highlights the important impact of selective logging on soil microbial C cycling not before demonstrated. As the exact age of logging gaps in this study was not known, and as they did not vary greatly in terms of forest regeneration, it was not possible to evaluate successional trajectories of soil microbial communities or recovery of microbial functions with time. We recommend future studies focus on soil microbial community attributes and biogeochemical cycling in logging gaps undergoing various stages of secondary succession to understand the legacy of logging disturbance. Evaluation and prediction of the impacts of selective logging should incorporate logging gap effects on belowground communities and processes, which may influence restoration potential and recovery of biodiversity and vital ecosystem services in human-modified tropical forests.

## Data Availability

The molecular dataset presented in this study in this study can be found in online repositories. The names of the repository/repositories and accession number(s) can be found below: https://www.ebi.ac.uk/ena, PRJEB71692.

## Footnotes

^1^ http://wdcpc.org
