# Supplementary material for: Selective logging impacts on soil microbial communities and functioning in Bornean tropical forest
Source: Front Microbiol. 2024 Sep 26;15:1447999. doi: 10.3389/fmicb.2024.1447999 (PMC11464443; doi:10.3389/fmicb.2024.1447999)
Supplement: Supplementary file 1 [file Data_Sheet_1.pdf]

Supplementary Material

1 Supplementary Materials and Methods

1.1 Molecular analysis of soil microbial communities

DNA was extracted from 0.1 g soil using the Quick-DNA™ Soil Microbe Kit and protocol (Zymo Research). Amplicon libraries were constructed according to a dual indexing strategy with each primer consisting of the appropriate Illumina adapter, 8-nt index sequence, a 10-nt pad sequence, a 2-nt linker and the amplicon specific primer (Kozich et al., 2013). For bacteria, V3-V4 16S rRNA amplicon primers (CCTACGGGAGGCAGCAG and GCTATTGGAGCTGGAATTAC) were used (Kozich et al., 2013). Fungi were targeted by amplifying the ITS2 region using primers GTGARTCATCGAATCTTTG and TCCTCCGCTTATTGATATGC (Ihrmark et al., 2012). Although concerns have been raised regarding the use of ITS primers for detecting AM fungi (Hart et al., 2015), more recent studies have demonstrated adequate identification of diversity and community patterns within sample types such as soil (Berruti et al., 2017; Lekberg et al., 2018). A high-fidelity DNA polymerase was used to generate amplicons (Q5 Taq, New England Biolabs). PCR conditions that followed an initial denaturation at 95 °C for 2 minutes were: denaturation at 95 °C for 15 seconds; annealing at 55 °C (16S) and 52 °C (ITS); annealing times were 30 seconds with extension at 72 °C for 30 seconds; cycle numbers were 25; a final extension of 10 minutes at 72 °C was included. An Agilent 2200 TapeStation system was used to determine amplicon sizes. Samples were normalized using a SequalPrep Normalization Plate Kit (Thermo Fisher Scientific) before pooling. The pooled library was quantified using a Qubit dsDNA HS kit (Thermo Fisher Scientific) prior to sequencing with an Illumina MiSeq using V3 600 cycle reagents at a concentration of 8 pM with a 5% PhiX Illumina control library. The sequencing run produced in excess of 21 and 18 million reads passing filter (16S and ITS, respectively). The DADA2 R package was used to quality filter, merge, de-noise and assign taxonomies to sequences (Callahan et al., 2016). 16S forward reads were trimmed to 250 bases. ITS sequence reads were trimmed to 225 and 160 bases, forward and reverse, respectively. Maximum number of Ns (maxN) = 0 and maximum number of expected errors (maxEE) = 1 were used as filtering settings. Sequences were dereplicated before applying the DADA2 core sequence variant inference algorithm. Forward and reverse reads were merged and chimeric sequences removed using the mergePairs and removeBimeraDenovo functions at default settings. Taxonomies were assigned to amplicon sequence variants (ASVs) using assignTaxonomy and the training database UNITE version 7.2 (UNITE Community, 2017).

1.2 Microbial biomass carbon (MBC) extraction method

MBC was determined using a modified chloroform fumigation extraction method (Brookes et al., 1985; Vance et al., 1987). Briefly, microbial biomass subsamples were passed through a 2 mm sieve to remove roots and stones. For each sampling point, two 2.5 g fresh weight soil were measured into separate 50 ml sample tubes. 12.5 ml 0.5M K2SO4 extractant was added to each sample tube (first adjusted to pH-neutral with NaOH) and gently hand shaken. 0.25 ml EtOH-free chloroform was then added to one sample. Sample tubes for fumigated and unfumigated samples were closed and shaken at approx. 300 rev min-1 for two hours on an orbital shaker. Sample tubes were centrifuged at 3000 rpm for 10 minutes, and the supernatant was filtered through Whatman no. 42 filter papers. Any remaining chloroform in fumigated extracts was removed by sparging with compressed air for 20

min. Total C content of extracts were measured using a Total Organic Carbon (TOC) analyser (TOC-L, Shimadzu Corporation, Kyoto, Japan). MBC was calculated as the difference between fumigated and unfumigated samples, expressed as µg g-1 dry soil after correction using soil moisture content. Clearly erroneous values (e.g. negative values) for two gap samples and one forest sample were removed from the final dataset.

1.3 Soil incubation method for heterotrophic respiration

Fresh weights of cores were recorded on removing rubber lids. Water holding capacity (WHC) was determined using one intact core from each sampling point and calculated using saturated weight after immersion in deionised water for 24 hours and oven dry weight (105˚ C for approx. 48 hours) (Öhlinger, 1995). The lower opening of all remaining cores were covered with plastic mesh and filter paper to prevent loss of material, before mounting on a plastic draining plate and placing upright in 1L Mason jars for incubation. Cores were separated into three experimental blocks (A, B and C) by sampling point, so that each block comprised three technical replicates of each plot, arranged in alternating CCF and SLG pairs (see fig below). Jars were covered with a moisture-resistant flexible film (Parafilm; Bemis, USA) punctured with air holes to reduce soil evaporation while avoiding anaerobic conditions.

A synthetic rain solution used for %WHC adjustment was prepared using autoclaved deionised water with additions of sodium chloride (NaCl: 0.29 g l-1), calcium chloride dihydrate (CaCl2.2H20: 0.09 g l-1), calcium sulphate dihydrate (CaSO4.2H2O: 0.07 g l-1), magnesium sulphate heptahydrate (MgSO4.7H2O: 0.13 g l-1) and sulphuric acid (98 % H2SO4: 0.23 g l-1) based on chemical composition of rain collected at nearby Danum Valley Field Station. Data were obtained from the World Data Centre for Precipitation Chemistry (http://wdcpc.org; Vet et al., 2014).

To determine rates of soil heterotrophic respiration, jars were sealed and CO2 concentrations measured using a Picarro gas analyser (Picarro Instruments, USA) arranged in a closed loop system with a measurement frequency of ~1 Hz. Increase in CO2 concentration were recorded for six minutes following an initial stabilisation period of approximately one minute. CO2 concentration data for each measurement were trimmed to the last 350 points to retain a period of linear increase over time, and rate of change in ppm was calculated using linear regression. CO2 efflux rates were then calculated in µg CO2-C cm-2 hour-1 using the following formula:

CO2 efflux rate = (3,600*m* V*CM*P)/(A*R*T)

where m is the rate of change in CO2 concentration (ppm s-1), V is the volume of chamber used during measurement corrected for soil core volume (m3), CM is the molecular mass of C (g mol-1), P is absolute gas pressure, A is surface area of core (m2), R is the universal gas constant and T is temperature (Kelvin).

1.4 References

Berruti, A., Desirò, A., Visentin, S., Zecca, O., and Bonfante, P. (2017). ITS fungal barcoding primers versus 18S AMF-specific primers reveal similar AMF-based diversity patterns in roots and soils of three mountain vineyards. Environ. Microbiol. Rep. 9, 658–667. doi: 10.1111/1758-2229.12574

Brookes, P. C., Kragt, J. F., Powlson, D. S., & Jenkinson, D. S. (1985). Chloroform fumigation and the release of soil nitrogen: The effects of fumigation time and temperature. Soil Biology and Biochemistry, 17(6), 831-835. https://doi.org/10.1016/0038-0717(85)90143-9

Callahan, B. J., McMurdie, P. J., Rosen, M. J., Han, A. W., Johnson, A. J. A., and Holmes, S. P. (2016). DADA2: high-resolution sample inference from Illumina amplicon data. Nat. Methods 13, 581–583. doi: 10.1038/nmeth.3869

Hart, M. M., Aleklett, K., Chagnon, P. L., Egan, C., Ghignone, S., Helgason, T., et al. (2015). Navigating the labyrinth: a guide to sequence-based, community ecology of arbuscular mycorrhizal fungi. New Phytol. 207, 235–247. doi: 10.1111/nph.13340

Ihrmark, K., Bodeker, I. T. M., Cruz-Martinez, K., Friberg, H., Kubartova, A., Schenck, J., et al. (2012). New primers to amplify the fungal ITS2 region - evaluation by 454-sequencing of artificial and natural communities. FEMS Microbiol. Ecol. 82, 666–677. doi: 10.1111/j.1574-6941.2012.01437.x

Kozich, J. J., Westcott, S. L., Baxter, N. T., Highlander, S. K., and Schloss, P. D. (2013). Development of a dual-index sequencing strategy and curation pipeline for analyzing amplicon sequence data on the MiSeq Illumina sequencing platform. Appl. Environ. Microbiol. 79, 5112–5120. https://doi.org/10.1128/AEM.01043-13

Lekberg, Y., Vasar, M., Bullington, L. S., Sepp, S. K., Antunes, P. M., Bunn, R., et al. (2018). More bang for the buck? Can arbuscular mycorrhizal fungal communities be characterized adequately alongside other fungi using general fungal primers? New Phytologist, 220, 971–976. doi: 10.1111/nph.15035

Öhlinger, R. (1995). Methods in soil physics: maximum water holding capacity. In F. Schinner, R. Öhlinger, E. Kandeler, & R. Margesin (Eds.), Methods in soil biology (pp. 385-386). Springer.

UNITE Community (2017). UNITE General Fasta Release. Version 01.12.2017.

Vance, E. D., Brookes, P. C., & Jenkinson, D. S. (1987). An extraction method for measuring soil microbial biomass C. Soil Biology and Biochemistry, 19(6), 703-707. https://doi.org/10.1016/0038-0717(87)90052-6

Vet, R., Artz, R. S., Carou, S., Shaw, M., Ro, C.-U., Aas, W., . . . Reid, N. W. (2014). A global assessment of precipitation chemistry and deposition of sulfur, nitrogen, sea salt, base cations, organic acids, acidity and pH, and phosphorus. Atmospheric Environment, 93, 3-100. https://doi.org/https://doi.org/10.1016/j.atmosenv.2013.10.060

2 Supplementary Figures

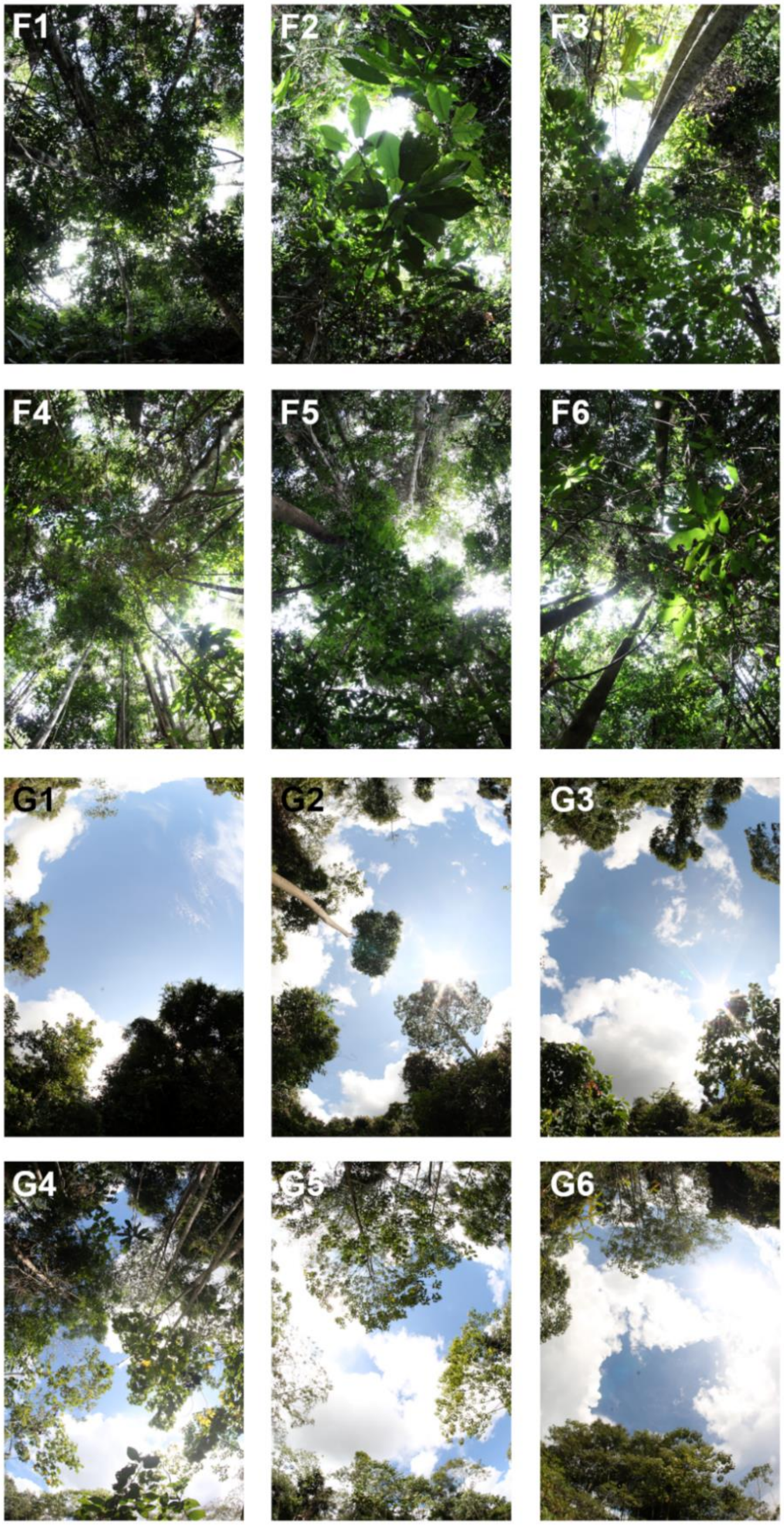

Supplementary Figure 1. Hemispherical canopy cover photographs taken at 2 m from ground level at the centre of each of the twelve 6 × 6 m study plots in intact closed canopy forest (F1-F6) and selective logging gaps (G1-G6).

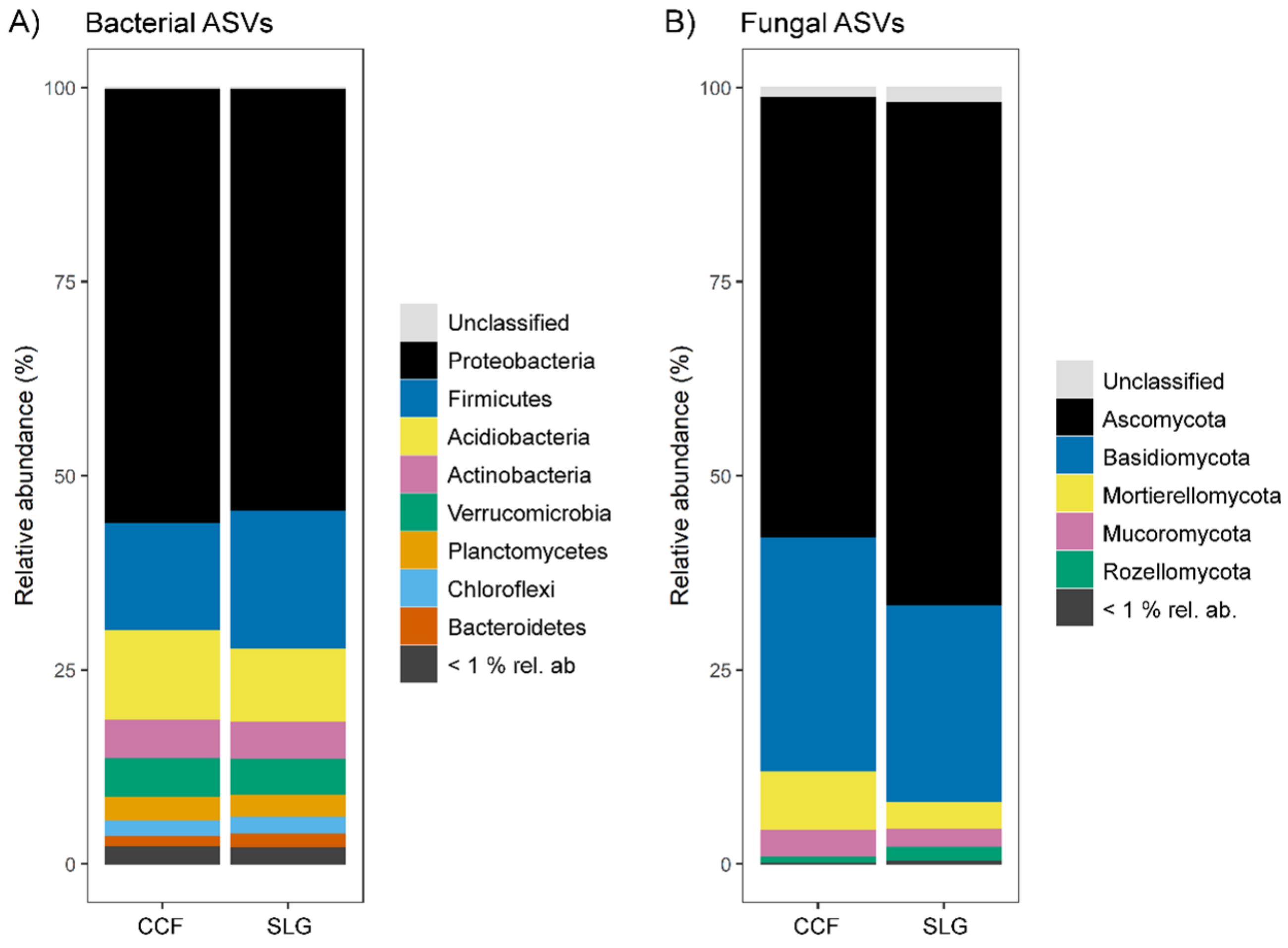

Supplementary Figure 2. Relative abundances of bacterial (**A**) and fungal (**B**) phyla as a percentage of total Amplicon Sequence Variants (ASVs) in intact closed-canopy forest (CCF) and selective logging gaps (SLG). Phyla with < 1% relative abundance in both vegetation types are represented as one group.

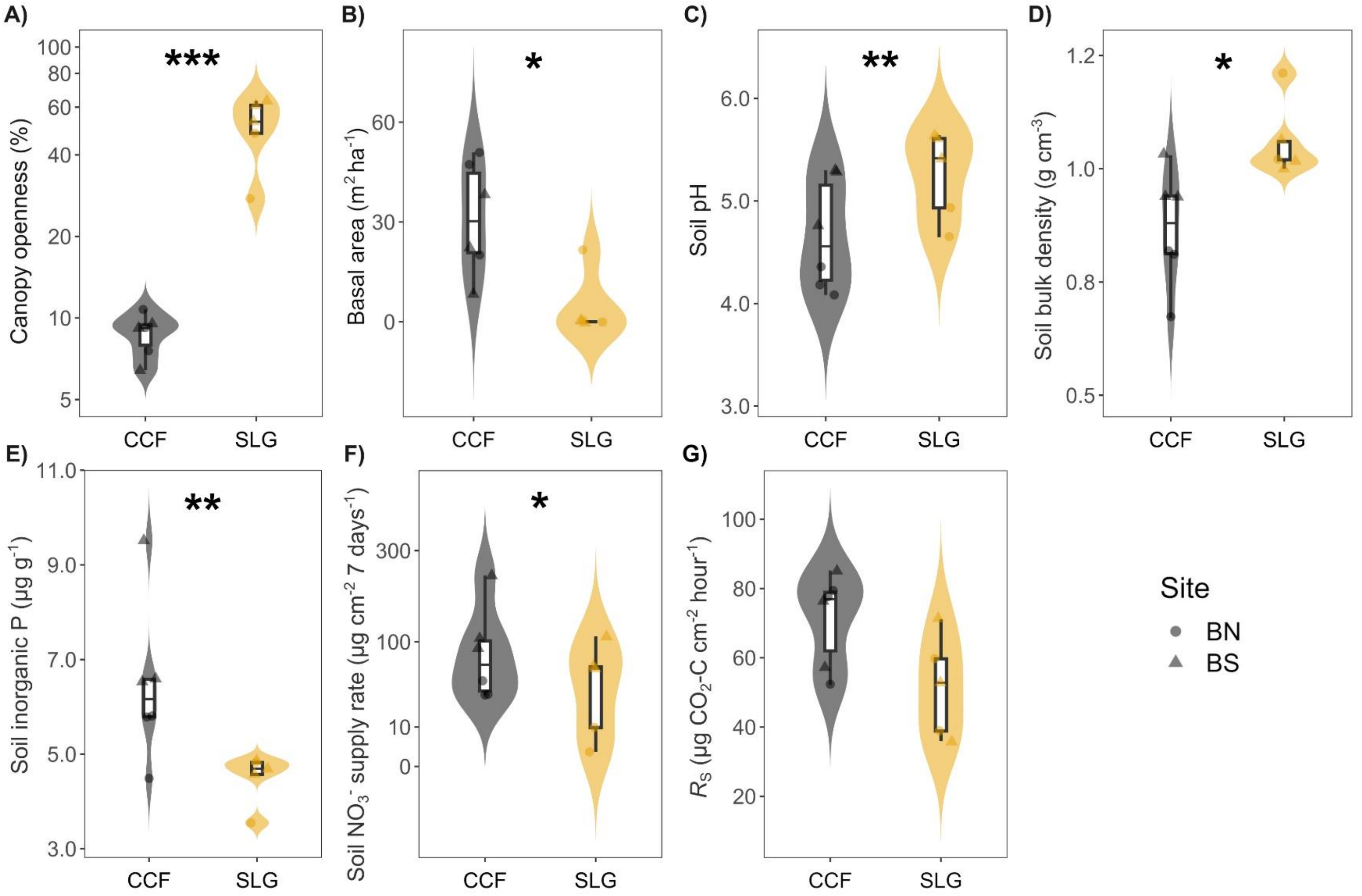

Supplementary Figure 3. Forest structural characteristics, soil properties and functions that significantly differed between intact closed canopy forest (CCF, grey) and selective logging gaps (SLG, orange) across the two study sites (B North, circles; B South, triangles) as identified with ANOVA after controlling for site. Canopy openness values **(A)** are presented on a log scale. R_S is soil heterotrophic respiration. Asterisks indicate significance level of statistical differences between vegetation types; * $p < 0.05$, ** $p < 0.01$, *** $p < 0.001$.

3 Supplementary Tables

Supplementary Table 1. Multiple linear regression results of soil characteristics explaining variation in fungal guild relative abundances. Variables are presented in order entered in the model. The factor 'Site' was entered first in all models to control for site effects. Partial R^2 represents the relative proportion of explained variance of each predictor, calculated from partitioned sums of squares. Corrected partial and model R^2 values represent proportion of explained variance of predictors after removing variance associated with the 'Site' categorical control variable. Continuous predictors were mean-centred and scaled by standard deviation to give standardised coefficients.

**Guild**	**Predictor**	***df***	**Partial R^2**	**Corrected partial R^2**	***F***	***p***	**Standardised coefficients**	**Corrected model R^2**
Mycorrhizal fungi	Site	1	0.04		1.00	0.351		0.68
	pH	1	0.41	0.43	9.29	0.019	-8.57	
	Total P	1	0.24	0.25	5.46	0.052	-9.89	
	Error	7						
EcM fungi	Site	1	0.03		0.68	0.438		0.70
	pH	1	0.44	0.45	10.68	0.014	-8.61	
	Total P	1	0.25	0.25	6.02	0.044	-9.69	
	Error	6						
AM fungi	Site	1	0.02		1.21	0.314		0.88
	pH	1	0.67	0.68	34.31	0.001	0.28	
	Organic layer depth	1	0.13	0.13	6.73	0.041	0.06	
	NH_4^+ supply rate	1	0.06	0.06	3.13	0.127	-0.07	
	Error	6						
Pathogenic fungi	Site	1	0.01		0.16	0.701		0.60
	pH	1	0.46	0.47	8.11	0.025	7.52	
	Total P	1	0.13	0.13	2.29	0.174	-4.24	
	Error	7						
Endophytic fungi	Site	1	0.05		0.89	0.378		0.61
	pH	1	0.49	0.52	9.41	0.018	1.29	
	C:N ratio	1	0.09	0.10	1.74	0.229	-0.49	
	Error	7						
Lichenised fungi	Site	1	0.31		42.74	0.003		0.96
	pH	1	0.42	0.61	56.86	0.002	-0.97	
	NO_3^- supply rate	1	0.09	0.13	11.65	0.027	0.35	
	P^- supply rate	1	0.08	0.12	11.38	0.028	-0.31	
	Organic layer depth	1	0.04	0.06	6.02	0.070	-0.22	
	NH_4^+ supply rate	1	0.02	0.03	3.08	0.154	-0.23	
	Error	4						
Saprotrophic fungi	Site	1	0.00		0.13	0.737		0.94
	Total P	1	0.80	0.80	53.09	0.002	12.17	
	Zn^+ supply rate	1	0.08	0.08	5.05	0.088	-1.82	
	C:N ratio	1	0.03	0.03	2.11	0.220	1.50	
	NH_4^+ supply rate	1	0.02	0.02	1.04	0.366	3.15	
	NO_3^- supply rate	1	0.01	0.01	0.89	0.399	-2.94	
	Error	4						
